# Immortalized murine fibroblast cell lines are refractory to reprogramming to pluripotent state

**DOI:** 10.18632/oncotarget.26235

**Published:** 2018-10-16

**Authors:** Elena V. Skvortsova, Sergey A. Sinenko, Alexey N. Tomilin

**Affiliations:** ^1^ Institute of Cytology, Russian Academy of Sciences, St Petersburg, Russian Federation; ^2^ Division of Molecular and Radiation Biophysics, B.P. Konstantinov Petersburg Nuclear Physics Institute, NRC “Kurchatov Institute”, Orlova Roscha, Gatchina, Russian Federation; ^3^ St Petersburg State University, St Petersburg, Russian Federation

**Keywords:** iPSC, NIH3T3, STO, pluripotency, cell reprogramming

## Abstract

To date different cell types of various mammalian species have been reprogrammed to induced pluripotent stem cells (iPSCs) using Yamanaka's cocktail of transcription factors (Oct4, Klf4, Sox2, and cMyc). It has been shown that several primary human cancer cell lines could be reprogrammed to iPSCs. We sought if immortalized mouse fibroblast cell lines could also be reprogrammed to iPSCs. The approach of generating iPSCs from such cells should be valuable in different experimental settings as it allows clonally derive cell lines carrying mutations whose impact on reprogramming could be next evaluated. Therefore, we investigated reprogramming of widely used immortalized cell lines (NIH3T and STO), as well as of *de novo* immortalized fibroblast line (tKM) with the use of highly effective lentiviral polycistronic OKSM expression system. Our reprogramming experiments have shown that in contrast to mouse embryonic fibroblasts (MEFs), none of the immortalized cell lines can be reprogrammed to pluripotent state. Contrary to colonies derived from MEFs, those derived from the immortalized cells lines (1) developed much later, (2) contained large round cells, not typical for iPSCs, and (3) were negative for trusted markers of matured iPSCs, Nanog and SSEA1. Immortalized cell lines NIH3T and STO are known to be mostly aneuploid, whereas tKM population includes cells with normal karyotype, however, neither cell type can be reprogrammed. Thus our data argue that aneuploidy *per se* is not a reason for the observed refractoriness of mouse immortalized cells to reprogramming to pluripotent state.

## INTRODUCTION

The major breakthrough in understanding mechanisms of maintenance and handling pluripotent stem cells become available after discovery of the method of reprogramming somatic cells to pluripotent state by four transcription factors, or so call Yamanaka`s cocktail: Oct4, Sox2, Klf4, and cMyc [1]. The reprogramming is mediated by resetting the epigenome of somatic cells and results in generation of induced pluripotent stem cells (iPSCs). They are functionally and molecularly analogous to embryonic stem cells (ESCs) originated from pre-implantation embryo epiblast [2, 3]. During reprogramming process, almost all cells go through the initiation phase characterized by the down-regulation of differentiation genes and the activation of early pluripotency factors. However, vast majority of cells remain refractory to reprogramming, and only rare cells go through the second stage, set up stable expression of the core pluripotency network, and become pluripotent [4, 5]. Although there is significant body of knowledge about molecular mechanisms of cell reprogramming to pluripotent state, yet these aspects remain poorly understood. To date different types of somatic cells were successfully reprogrammed to pluripotent state [6–13]. iPSCs of different mammalian species including primates, rodents, ungulates, and felines were generated with use of standard reprogramming technique [14–20]. However there are some differences in a way of applying the reprogramming method to different cell types and cells from different species. For instance, in contrast to mouse fibroblasts, rat fibroblasts can be reprogrammed only in serum-free media conditions [16]. Reprogramming efficiency depends on stochastic equilibrium and levels of expression of pluripotency transcription factors. Recent data support deterministic versus stochastic model of cell reprogramming [21].

To date there were multiple attempts to reprogram malignant cancer cell lines. It was shown that a number of human primary cancer cells lines of different origin could be reprogrammed to pluripotent state [22–27]. However, generation of cancer-derived iPSCs remains a challenging task. The reprogramming inefficiency and relative instability of the cancer-derived iPSCs became the main issues [28, 29]. On the other hand, referring to the published data, iPSCs have been generated only from few mouse primary cancer cell lines, suggesting that mouse cancer cells are mainly refractory to reprogramming in standard condition [29, 30]. It is known that multiple passages and culture conditions lead to significant abnormalities in chromosome numbers and stability. Efficiency of cell reprogramming significantly decreases at advanced passages of primary cell cultures and in more terminally differentiated cells [31, 32]. However it has not been yet analyzed and understood how prolonged cultivation leads to refractoriness to the reprogramming. On the other hand, malignant transformed or immortalized cell lines have several advantages over primary cell cultures, such as easy handling, high proliferation rate, and clonogenicity, all are beneficiary in several experimental setups. In particular, use of such cells is beneficial because it would allow to clonally derive mutants which could be subsequently assayed for reprogramming to iPSCs. Here we provide an experimental evidence of inability of immortalized cells, both aneuploid and normal, to be reprogrammed into iPSCs, indicating that aneuploidy is not the cause of such refractoriness.

## RESULTS AND DISCUSSION

### OKSM cassette is more efficient than OSKM for cell reprogramming into iPSCs

**Figure 1:**
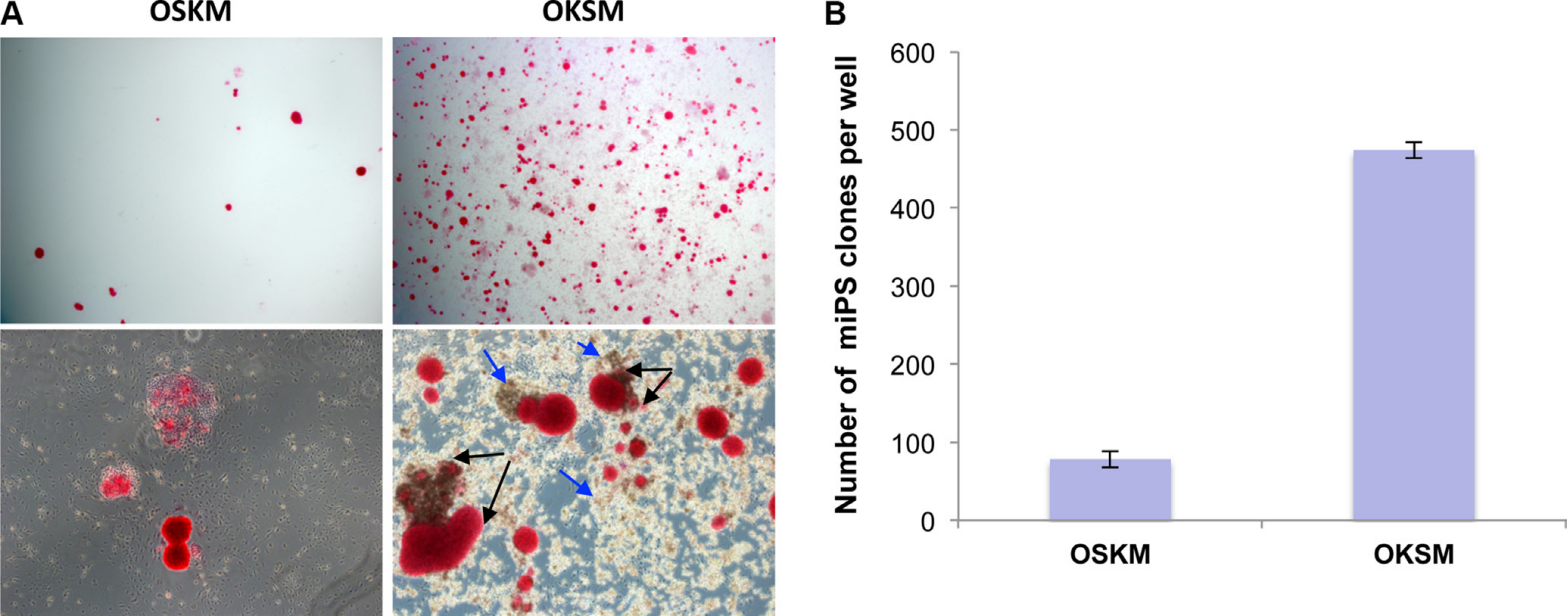
OKSM polycistronic vector is more efficient in generation of iPSCs (**A**) iPSC clones revealed by alkaline phosphatase (AP) staining on day 14 following infection with polycistronic lentiviruses OSKM or OKSM; magnifications: 4x – upper images, 10x – lower images. Presumable sister iPSC clones within the clusters indicated by black arrows. Conglomerates of large round-shaped “intermediate” cells indicated by blue arrows. (**B**) Counts of AP-positive iPSC clones generated by day 14 with the use of OSKM or OKSM cassettes; results are expressed as mean ± SD, *n* = 3.

To reprogram mouse embryonic fibroblasts, we used two previously developed vectors bearing all four reprogramming transcription factors. The OSKM vector representing single polycistronic vector allowing expressing full-length murine Oct4, Sox2, Klf4, and cMyc by autonomous “self-cleaving” 2A peptides [33]. Another polycistronic vector expressing full-length human Oct4, Klf4, Sox2, and cMyc (OKSM) interspaced with the 2A peptides and IRES sequence was also used [34]. Polycistronic vectors are more efficient in reprogramming and besides, allow to ectopically express one copy of each gene per cell [33]. Almost complete homology between these transcription factors in mammals allows using them for reprogramming of both human and mouse cells. First, we performed reprogramming of MEFs with the use of each of the two lentiviral constructs. We have found that OKSM construct shows about 60-fold higher reprogramming efficacy (6%) vs. OSKM (0,1%) (Figure 1). The cause of this dramatic difference in reprogramming efficacy is not clear, given titers of both polycistronic viruses and multiplicity of infection (MOI) are approximately equal, however, it can be assumed that ratios between expression levels of the four transcription factor is critical. For instance, it was shown that efficiency of reprogramming is much higher when levels of expressions of Oct4 and Klf4 significantly exceed those of cMyc and Sox2 [35, 36]. Interesting to note that in comparison to OSKM, transduction of MEFs with OKSM virus led to development of primary or intermediate clones (starting on day 7), consisting of relatively large round-shaped cells (Figure 1, and Supplementary Figure 1, indicated by arrows). These cells probably represent an intermediate stage during cell reprogramming. It was also obvious that true MEF-derived iPSC clones (at day 14) often positioned as clusters of colonies of different sizes (Figure 1, and Supplementary Figure 1, depicted with arrows). This might indicate that these clusters of clones are likely to derive from one parental cell. In this scenario one single fibroblast is reprogramed to “intermediate” iPSCs colonies with round-shaped cells, and some of these cells are further reprogrammed to *bona fide* iPSCs, forming the observed clusters of colonies. This is consistent with an observation that fast-cycling cells give increased cell numbers and they likely have certain intrinsic properties, such as epigenetic predisposition to being reprogrammed [21]. Previously, it was reported that OKSM STEMCCA polycistronic cassette was highly efficient in iPSCs generation while the large number of clones induced by this construct displayed lack of Nanog expression [37]. Importantly, we used another OKSM construct [34], which induced a high number of iPSC clones, and all of these clones expressed high levels of Nanog (see below). We have also found that N2B27 2i serum-free media is more reproducible and efficient than serum-based media for iPSCs generation (data not shown). The OKSM polycistronic vector and N2B27 2i media were selected for further cell reprogramming experiments.

### NIH3T3 and STO cells cannot be reprogrammed to iPSCs

**Figure 2:**
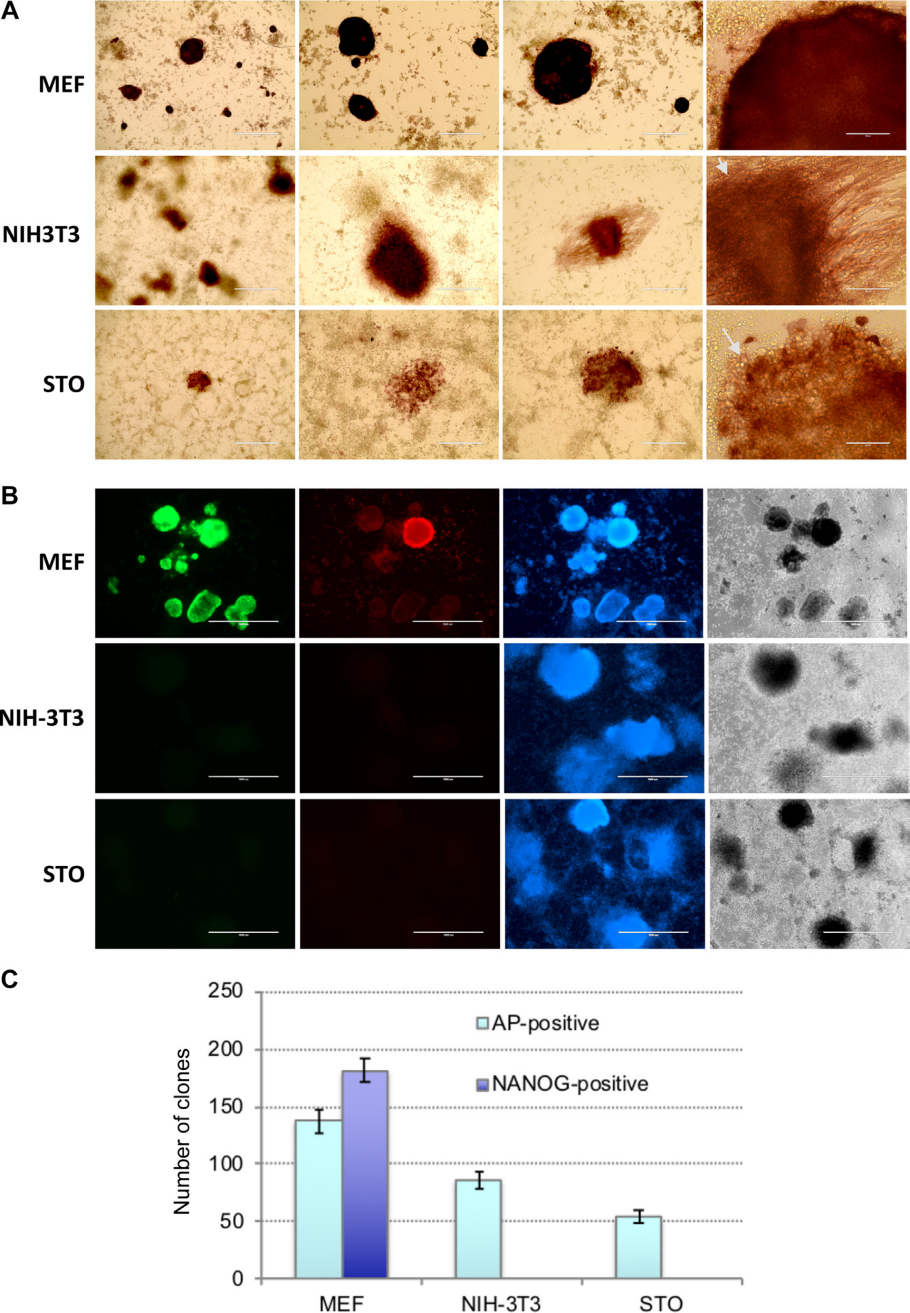
NIH3T3 and STO cells cannot be reprogrammed into iPSCs (**A**) Representative images of cell clones derived from MEFs, NIH3T3, and STO cells, as revealed by AP-staining on day 14 after OKSM lentivirus infection; large round shaped cells indicated by arrows. (**B**) Immunostaining of the colonies on day 14 after OKSM induction revealed lack of Nanog (green) and SSEA1 (red) expression in colonies derived from NIH3T3 and STO cells, contrary to those derived from MEFs; cells were counterstained with DAPI (blue) (**C**) Counts of AP-positive and Nanog-positive cell colonies derived from MEFs, NIH3T3, and STO cells generated on day 14 after OKSM lentivirus infection; results are expressed as mean ± SD, *n* = 2.

It is often highly desirable to assess roles of genes of interest in reprogramming to iPSCs, applying CRISPR/Cas9 or more traditional methods of transgenesis to cells prior to reprogramming. However, a vast majority of primary cell types used for reprogramming, such as MEFs or blood cells, have limited proliferation potential, and thus, derivation of mutant clones for subsequent iPSC derivation assays is not feasible. On the contrary, immortalized or transformed cells of established cell lines posses basically unlimited clonogenic potential. Therefore, we attempted to reprogram to iPSCs widely used mouse cell lines of fibroblast origin, namely NIH3T3 and STO. To this end, we used the above OKSM polycistronic vector which showed superior reprogramming efficiency. MEFs, NIH3T3, and STO cells were transduced with equal amounts of viruses. Important to note that NIH3T3 and STO cells proliferated significantly faster than MEFs, i.e. >2.5 times (Supplementary Table 1). Round-shaped clones have been developed in NIH3T3 and STO cell cultures starting from day 9. Cells within these clones were round and different from regular iPSCs (Figure 2A, indicated by arrows). Majority of those clones were positive for alkaline phosphatase (Figure 2A). Immunostaining for pluripotency markers Nanog and SSEA1 revealed that none of these clones expressed the proteins, which is opposed to MEF-derived iPSC clones (Figure 2B, 2C, see details in Material and Methods). These results suggests that OKSM is able to trigger the process of cell reprogramming, evidenced by developed primary clones, however, the latters fail to further proceed to pluripotent state. Three independent reprogramming experiments showed no signs of iPSC generation from NIH3T3 and STO cells. These cell lines could not be reprogrammed to iPSCs using either OSKM, or mixture of Oct4, Sox2, Klf4, cMyc viruses (Supplementary Figure 2). We also attempted to culture several clones derived from NIH3T3 and STO cells. Expectedly, 15 and 10 selected clones derived from each of these cell lines could not be maintained as iPSCs in mouse embryonic stem cell media. All these cells showed a typical morphology of differentiated cells that resembled fibroblasts (Supplementary Figure 3). We also observed that mouse cell line OP9, which represents immortalized embryonic bone morrow stromal stem cell origin, cannot be reprogrammed to iPSCs with OKSM (Supplementary Figure 4). It is known that all studied immortalized cell lines have chromosomal abnormalities. Most karyotypically characterized NIH3T3 cell line has 98,8% aneuploidy where 60% of cells are tetraploids with numerous chromosomal translocations, including balanced and unbalanced translocations, inverted duplications, deletions, or complex rearrangements [38]. STO and OP9 cell lines are also aneuploids, however, the degree of the karyotype abnormality has not yet been evaluated in details [39, 40]. Another feature of immortalized cell line is high proliferative index and constitutive activation of signaling pathways mediating high proliferation rates. It is likely that these two features are the major causes of the refractoriness to the cell reprogramming, however, this hypothesis requires further corroboration. Besides, it is likely that there is a significant reduction of p53 function in immortalized cell lines, however, while loss of p53 is advantageous for iPSC generation from primary cells [41, 42], it does not seem to predispose immortalized cells to better reprogramming.

### Newly established cell line tKM cannot be reprogrammed to iPSCs

**Figure 3:**
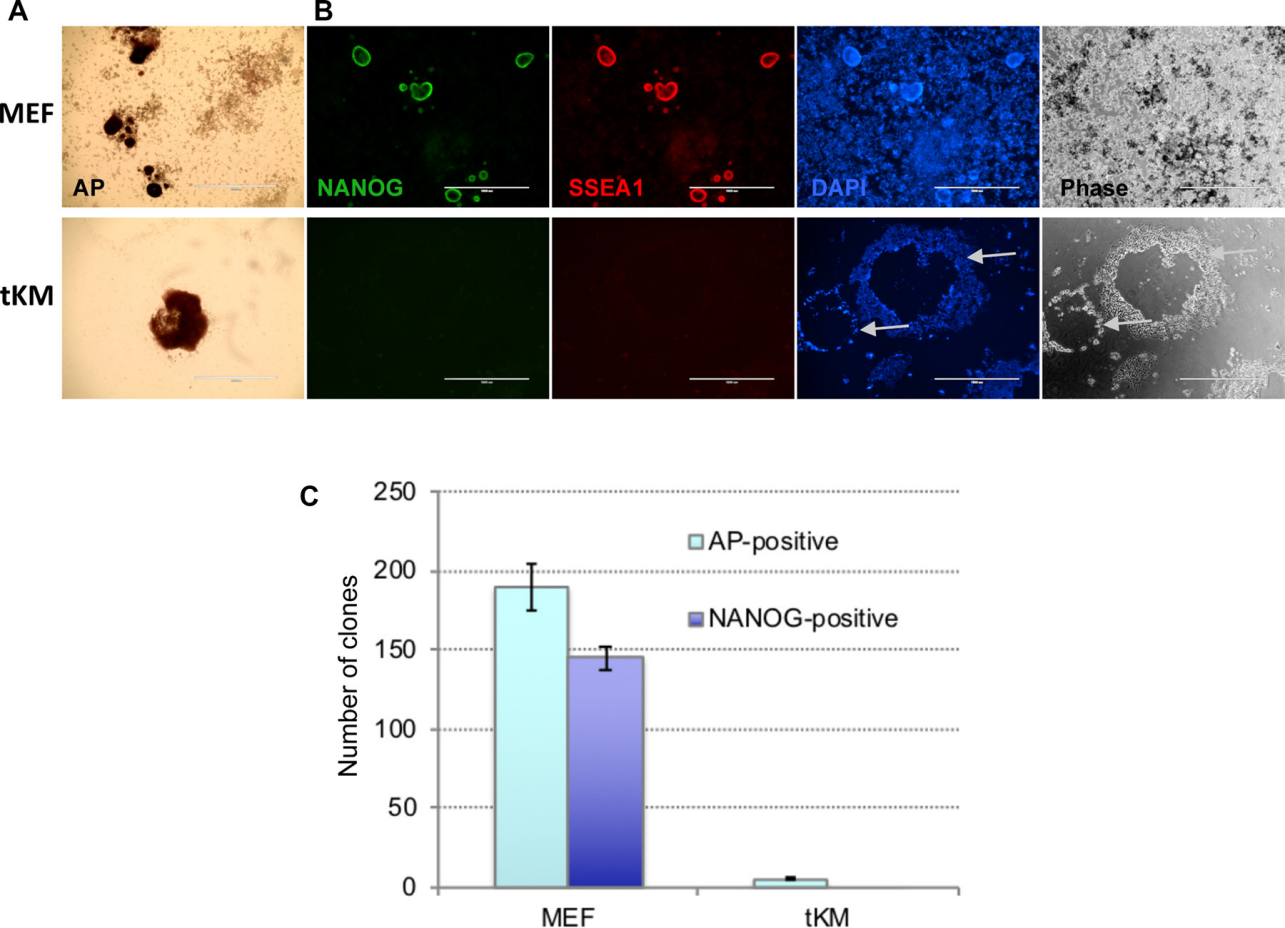
*De novo* immortalized fibroblasts (tKM cells) cannot be reprogrammed to iPSCs Representative images of cell colonies derived from tKM cells and, for control, MEFs, revealed by AP-staining (**A**) or by immunostaining for Nanog (green) and SSEA1 (red) (**B**) on day 14 after OKSM lentivirus infection. Cells were counterstained with DAPI (blue). tKM-derived clones are rare, notably larger, and have very low adhesion to plastic surface, compared to MEF-derived clones. (**C**) Counts of AP- and Nanog-positive cell colonies derived from MEFs and tKM cell; results are expressed as mean ± SD, *n* = 2.

It is known that long time exploited cell lines such as NIH3T3 and STO accumulate multiple chromosomal abnormalities and evolve dramatically to maintain themselves through numerous passages in culture. We asked whether immortalized cells with normal karyotype can be reprogrammed to pluripotency. To this end, we first infected MEFs with lentiviruses encoding Klf4, cMyc, and the repressor tT-KRAB, then selected for blasticidin S-resistant clones in the presence of Dox (to keep tT-KRAB away from repressing the Klf4, cMyc viruses). One of the picked clones, referred hereafter to as tKM, could be subsequently passaged in the absence of Dox, which ensured lack of the exogenous Klf4 and cMyc expression. Karyotypic analysis revealed that after multiple passages (>18) 56% of tKM cells preserved normal karyotype i.e. 40 chromosomes (Supplementary Figure 5 and Supplementary Table 2). Similar to NIH3T3 and STO, tKM cells infected with OKSM at passage 19 could give rise to some AP-positive colonies, however, all of them were negative for Nanog and SSEA1. Besides, these colonies were significantly larger than MEF-derived iPSC colonies, they became rather loose, easily detached from plate surface (Figure 3). Also, after picking the tKM-derived colonies, they could not be further maintained and passaged like iPSC in embryonic stem cell media. Thus, tKM fibroblasts, which have mostly normal ploidy, are refractory to reprogramming, like mostly aneuploid NIH3T3 and STO cells. This result suggests that aneuploidy *per se* is not the factor that counteracts OKSM-mediated reprogramming into iPSCs.

Several other reports also showed refractoriness of mouse cancerous cell lines to the reprogramming [3, 43]. At the same time, it was shown that primary mouse carcinoma cells and malignant melanoma cell line remain amenable to reprogramming to iPSCs [10, 29, 30]. Also it was shown that immortalized mouse fibroblast cells m5S can be efficiently reprogrammed via cell fusion with ESCs [44]. The two situations, however, cannot be compared one-to-one, because mechanism of cell fusion-based reprogramming might be significantly different from reprogramming driven by OKSM [21, 44, 45].

What could be the factors serving as roadblocks on the way immortalized cell being reprogrammed into iPSC? It is known that an abnormal cell signaling, for instance, activation of MAPK (p38) [46–48], TGFβ [49, 50], or Hippo/LATS2 [51] pathways strongly suppress the iPSC generation. From the other side, it was shown that cell immortality is a crucial and rate-limiting step towards the establishment of a pluripotent state in somatic cells. Such cells with low p19 protein levels as well as immortal fibroblasts deficient in components of p53 pathway develop iPSCs colonies much faster and almost each of these somatic cells have the potential to form iPSCs [52]. Activation of p16 (Ink4a) and p19 (Arf) promotes p53-p21 signaling, inhibiting iPSC generation [41, 42]. Activation of AMP-activated protein kinase (AMPK) provides a metabolic barrier to reprogramming somatic cells into stem cells [48, 53, 54]. Another relevant example is spermatogonial stem cells (SSCs) which are refractory to reprogramming to pluripotency [55]. Compared to ESCs, SSCs express lower levels of Oct4 and Sox2, and higher levels of Klf4 and cMyc. Deregulated expression of these pluripotency genes triggered by ectopic overexpression of OSKM and abnormal epigenetic regulation of specific regulatory elements involved in reprogramming might be the cause of the refractoriness of these cells to iPSC reprogramming. Moreover, it was shown that p53 pathway does not contribute to the refractoriness of SSCs to reprogramming [55]. It was also shown that naked mole rat (NMR) fibroblasts have drastically reduced propensity for reprogramming, compared to mouse fibroblasts. Inactivation of Rb alone, but not of p53, or expression of SV40 LT-antigen was sufficient to improve reprogramming efficiency. Compared to mouse, NMR had higher levels of repressive H3K27 methylation marks and lower levels of activating H3K27 acetylation marks, indicative of a more stable epigenome that resisted cell reprogramming [15].

Important to note that human primary cancer cell lines could be reprogrammed to iPSCs more efficiently than mouse ones [22, 26, 29, 56–60]. Several studies have reported generation of iPSCs from primary chronic myeloid leukemias, MLL-AF4-overexpressing hematopoietic stem cells/B progenitors, indicating that B-cell origin and leukemic fusion gene were not reprogramming barriers [28, 58, 61]. It still remains an unresolved question whether success of reprogramming depends only on the presence of so call “elite” cells, or it can be reached by any cell [21, 62]. Resent studies applying cellular barcoding technique indicate deterministic versus stochastic model of reprogramming [21]. The authors also showed that a population of fast-cycling cells was characterized by a high reprogramming potential. Although the investigated cell lines have high proliferation rate, the rates of generation of pseudo-iPSC clones from this cells are not higher than for MEFs (Supplementary Table 1). It is assumed that reprogramming potential is inherent to particular cell type and could be passed on through cell division. It is well known that less differentiated somatic precursor cells are more efficiently reprogrammed to pluripotency [63]. One can assume that long time maintained cell lines are evolved in more differentiated cell state that are refractory to reprogramming. Our experiments have shown that while the initial step of reprogramming triggered by OKSM occurs in NIH3T3, STO, and tKM cells, they could not be further reprogrammed to pluripotent state. Previous reports indicated that chromosomal abnormalities, and aneuploidy, as well as altered cell signaling associated with the cancer cell lines is a cause of the inability to be reprogrammed to pluripotent state [57, 64, 65]. Our analysis indicates that among these features aneuploidy of the studied murine cell lines is unlikely to be a roadblock to pluripotency reprogramming. Further analysis of mouse cancer cell lines in terms of pluripotency reprogramming will help to decipher the exact mechanism of their resistance to this process.

## MATERIALS AND METHODS

### Lentiviruses

The LVTHM-T7-Oct4, -Sox2, -cMyc, and -Klf4 constructs were described elsewhere (Liskovykh et al., 2011). LV-tTR-KRAB-iresBsd was derived from LV-tTR-KRAB-iresDsRed [66] by replacing the DsRed sequence with blasticidin S resistance gene. Other constructs were kindly provided by different labs: LV-tTR-KRAB-dsRed, pMD2.G, and psPAX2 [66], HAGE2-TetO-miniCMV-hOct4-F2A-hKlf4-IRES-hSox2-E2A-hcMyc-W-loxP (OKSM) [67], tetO-FUW-OSKM (OSKM, Addgene plasmid #20321) and FUW-M2rtTA (M2rtTA, Addgene plasmid # 20342) [33]. Lentiviruses were packaged in 293T cells using polyethylenimine hydrochloride (PEI 40 kDa, 40 μg) as a transfection method [68]. Lentivirus particles in cell culture supernatant were collected, concentrated to 5−10 × 10^6^ TU/ml as described elsewhere [16, 66, 69, 70].

### Cell lines

Mouse embryonic fibroblast derived cell lines, NIH3T3 and STO, were obtained from the Russian Cell Culture Collection (Institute of Cytology RAS, St Petersburg, Russia) where they had been karyotyped, as follows: 65–73 chromosomes (75–90% of cells), 1–2% microchromosomes, and 1.2% of polyploid cells for NIH3T3, 55–65 chromosomes, 1–2 microchromosomes, and 7.0% of polyploid cells for STO [38, 40]. Embryonic bone morrow stromal stem cell line OP9 were obtained from ATCC (CRL2749). Murine embryonic fibroblasts (MEFs), prepared from 13.5–14.5 dpc C57Bl6 mouse embryos [16], were infected with the lentiviruses LVTHM-T7-Klf4, LVTHM-T7-cMyc, and pLV-tT-KRAB-iresBsd, then cultured for 12 days in presence of Doxycycline (Dox, 5 μg/ml) and blasticidin S (2 μg/ml). Several resistant colonies were picked, cultured for 2–3 passages in the same medium, then cultured for several passages in Dox-free medium. One clone, referred to as tKM, which showed robust proliferation in the absence of Dox was considered immortalized and thus, was proceeded to iPSC derivation experiments.

### Reprogramming mouse fibroblasts with OKSM or OSKM/M2rtTA

MEFs, NIH3T3, and STO cells were grown in standard MEF media containing DMEM media (Biolot, Russia), 10% FBS (HyClone), 100 U/ml penicillin, 100 μg/ml streptomycin, and 2 mM l-glutamine (Gibco). Cells were treated against mycoplasma by culturing with 10 μg/ml ciprofloxacin (Myco-3, AppliChem, EU) or 10–25 μg/ml Plasmocin (Invivogen) in media for 1–2 passage. Monitoring mycoplasma was routinely performed by PCR. Cell cultures were maintained at 5% CO_2_ at 37°C. Cells were seeded (30 × 10^3^ cells per well) on 0.1% gelatin-coated 24-well plate in the MEF medium. Next day media was replaced with 200 μl of Opti-MEM media (Gibco) containing mixture of lentiviruses (MOI = 2–5 for each): (1) M2rtTA + pHAGE2-OKSM, (2) tetO- OSKM + M2rtTA, or (3) LVTHM-T7-Oct4, -Sox2, -Klf4, -cMyc (see *Lentiviruses* for details). Cells were incubated with the virus mixtures for 3–4 hrs, then 200 μl of Opti-MEM were added and incubation was continued overnight. Next day media was changed to MEF media containing 3 μg/ml Dox. Media was changed to fresh every day and on the 3rd day cells were trypsinized and seeded onto wells of 12- or 6- well plates pre-coated with gelatin and feeder cells (mitomycin C-treated MEFs) and cultured in N2B27 2i media containing standard N2B27 medium (Gibco) supplemented with 3 μM CHIR-99021 (Axon Medchem) 1 μM PD-0325901 (Axon Medchem), recombinant hLIF (5 ng/ml), and 3 μg/ml Dox at 37°C in a standard CO_2_ incubator. Medium was changed every next or second day. Clones became visible on day 9, and on day 14–15 they were fixed and proceeded for immunostaining. To ensure reproducibility, all reprogramming experiments were repeated at least twice, and results from one of these experiments were presented on each figure (Figures 1–3). Standard deviations between repeats (wells repeats) within the particular experiment are shown on the diagrams. We noticed that in some experiments with MEF reprogramming the number of Nanog-positive clones exceeded the number of AP-positive clones (Figure 2C). This variability in clone counts was primarily because often Nanog immunostaining revealed multiple clones within clone cluster that was recognized as one large AP-positive clone. We represented both cases of the experiments on Figures 2C and 3C.

### Immunostaining

Mouse iPSCs colonies growing on culture plates were fixed with 4% paraformaldehyde (PFA) in PBS (10 min, at room temperature), washed with PBS and treated for 30 min with blocking solution: 1% BSA, 2% non-immune sheep serum, 0.1% Tween-20 in PBS. Fixed cells were incubated with antibodies to mouse Nanog (Bethyl) or Anti-SSEA1 (Developmental Studies Hybridoma Bank), followed by corresponding secondary antibodies conjugated with Cy-3 or FITC (Jackson ImmunoResearch). Afterwards, wells were washed in 0.1% Tween20-PBS, counterstained with DAPI, and embedded under coverslips into an anti-fading media.

### Alkaline phosphatase staining

Mouse iPSC colonies were fixed with PFA and stained for alkaline phosphatase (AP) as described [16] with some minor modifications. PFA-fixed clones were washed in PBS and 25 mM Tris-maleate buffer (pH 9.0) and then incubated with the substrate mixture containing Tris-maleate buffer (pH 9.0), 4 mM MgCl_2_, 0.2 μg/ml 1-Naphthyl phosphate disodium salt (Sigma), and 0,5 μg/ml Fast Red TR Salt (Sigma), or SigmaFast Fast Red TR/Naphthol AS-MX Tablets (Sigma-Aldrich). The reaction was stopped by washing with PBS.

### Ethics statement

All animal studies were performed in accordance with the relevant guidance and regulations from the Interinstitutional Bioethical Committee of the Institute of Cytology of the Russian Academy of Sciences, Saint Petersburg, Russia.

## SUPPLEMENTARY MATERIALS FIGURES AND TABLES


