# Supplementary material for: Immortalized murine fibroblast cell lines are refractory to reprogramming to pluripotent state
Source: Oncotarget. 2018 Oct 16;9(81):35241–50. doi: 10.18632/oncotarget.26235 (PMC6219659; doi:10.18632/oncotarget.26235)
Supplement: Supplementary file 1 [file oncotarget-09-35241-s001.pdf]

Immortalized murine fibroblast cell lines are refractory to reprogramming to pluripotent state

SUPPLEMENTARY MATERIALS

SUPPLEMENTARY METHODS

Preparation of metaphase spreads and chromosome numbers scoring

Metaphase spreads were prepared as described previously [1] with modifications. tKM cells growing at logarithmic phase (cell confluence 60–80%) were treated for 10 hours or overnight with 0.4 µg/ml Colcemid (Wako Pure Chemical) at 37°C in 5%CO_2 incubator. Cells were harvested and incubated in hypotonic 0.56% KCl solution for 20 minutes. Then, cells were fixed by solution of methanol/acetic acid (3:1, v/v), washed, and stored in the fixative solution at −20°C. For metaphase spreads, cells suspension was placed drop wise on microscope glass slides (Superfrost; Thermo Scientific, Darmstadt, Germany), air-dried, and kept one week at room temperature (RT) on air. Metaphase spreads were stained by Giemsa and scored by phase contrast imaging light microscopy (EVOS FL Auto Imaging System).

REFERENCES

1. Liskovykh M, Ponomartsev S, Popova E, Bader M, Kouprina N, Larionov V, Alenina N, Tomilin A. Stable maintenance of de novo assembled human artificial chromosomes in embryonic stem cells and their differentiated progeny in mice. Cell cycle. 2015; 14:1268–1273.

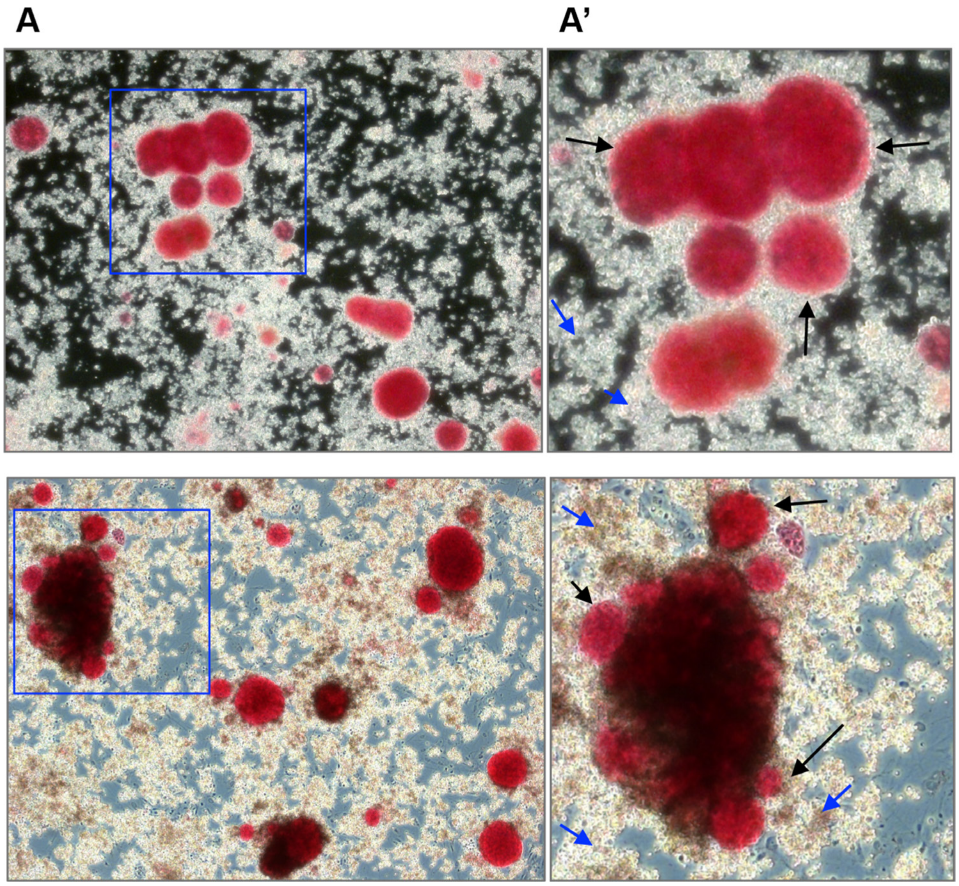

Supplementary Figure 1: Clusters of MEF-derived iPSC clones generated with OKSM construct. Representative images of reprogramming cell clones derived from MEFs, presumable sister iPSC clones within the clusters indicated by black arrows. Conglomerates of large round-shaped “intermediate” cells indicated by blue arrows. Magnification 10x (**A**) and 40x (**A’**) indicated on images.

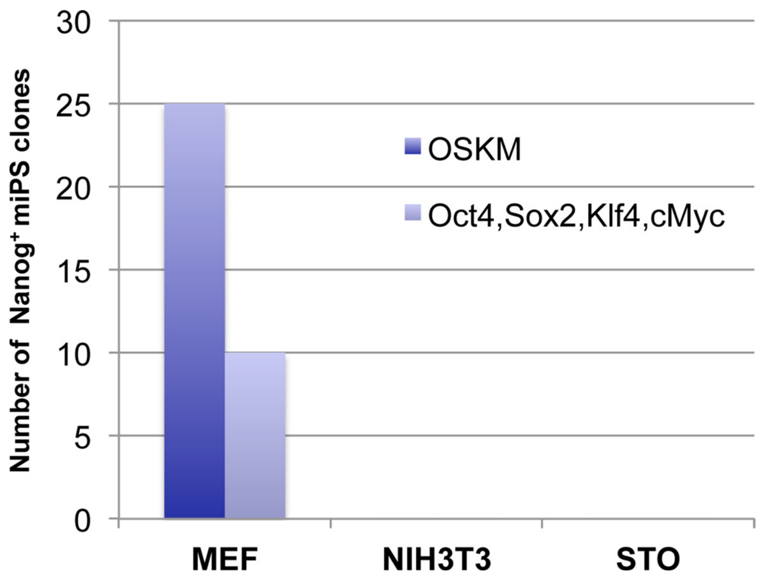

Supplementary Figure 2: NIH3T3 and STO cells could not be reprogrammed into iPSCs with the use of OSKM or mixture of single constructs of reprogramming factors. MEFs are efficiently reprogrammed by OSKM vector or by mixture of single constructs encoding the reprogramming factors. In contrast NIH3T3 and STO cells could not be reprogrammed to iPSCs by these constructs.

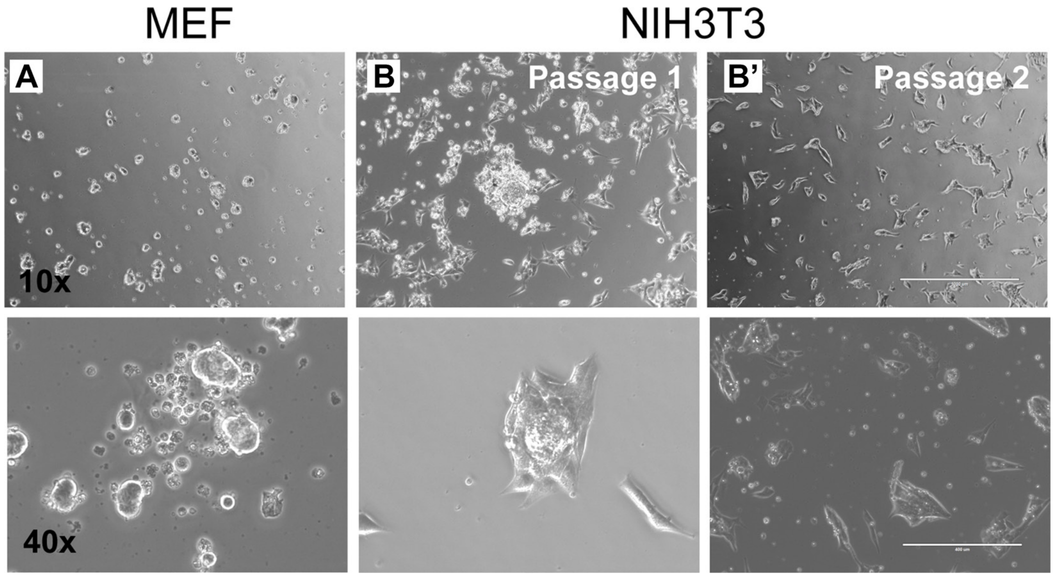

Supplementary Figure 3: Lack of iPSC maintenance of NIH-3T3-derived clone. Representative images of reprogramming cell clones derived from NIH-3T3 cells after 1st (**B**) and 2nd (**B'**) passages in compare with iPSC clones derived from MEFs (**A**). Magnification 10x and 40x indicated on images.

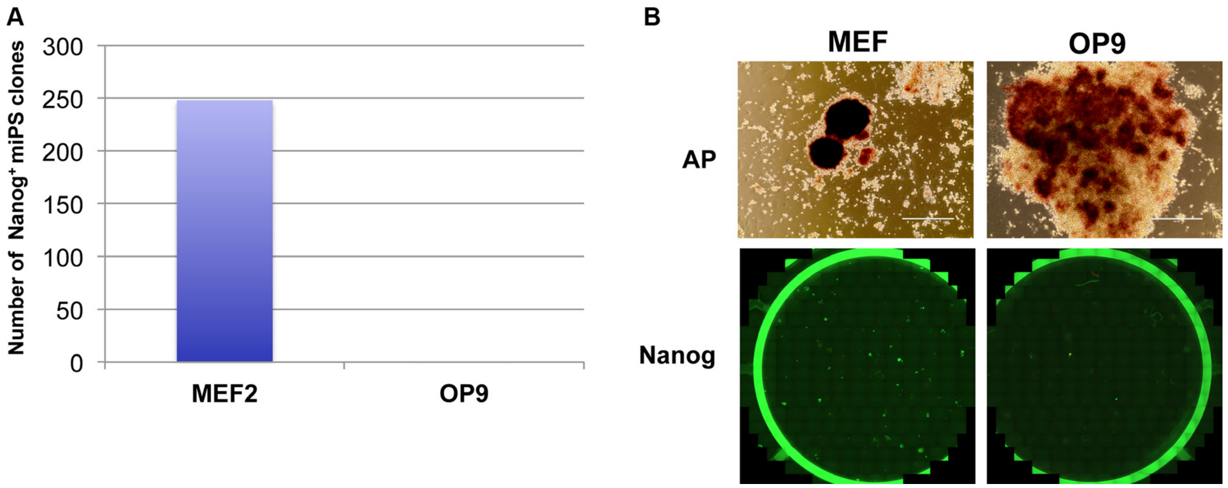

Supplementary Figure 4: (**A**) OP9 cell line could not be reprogrammed into iPSCs by OKSM as seen by absence of Nanog positive clones. (**B**) Representative view of cell colonies derived from MEFs and OP9 cells stained for alkaline phosphatase (10x magnification). Representative view culture wells containing Nanog-positive iPSC colonies (green) derived from MEFs, and lack of Nanog-positive colonies derived from OP9 cells (two green out of focus dots are pseudo signals, 2x magnification).

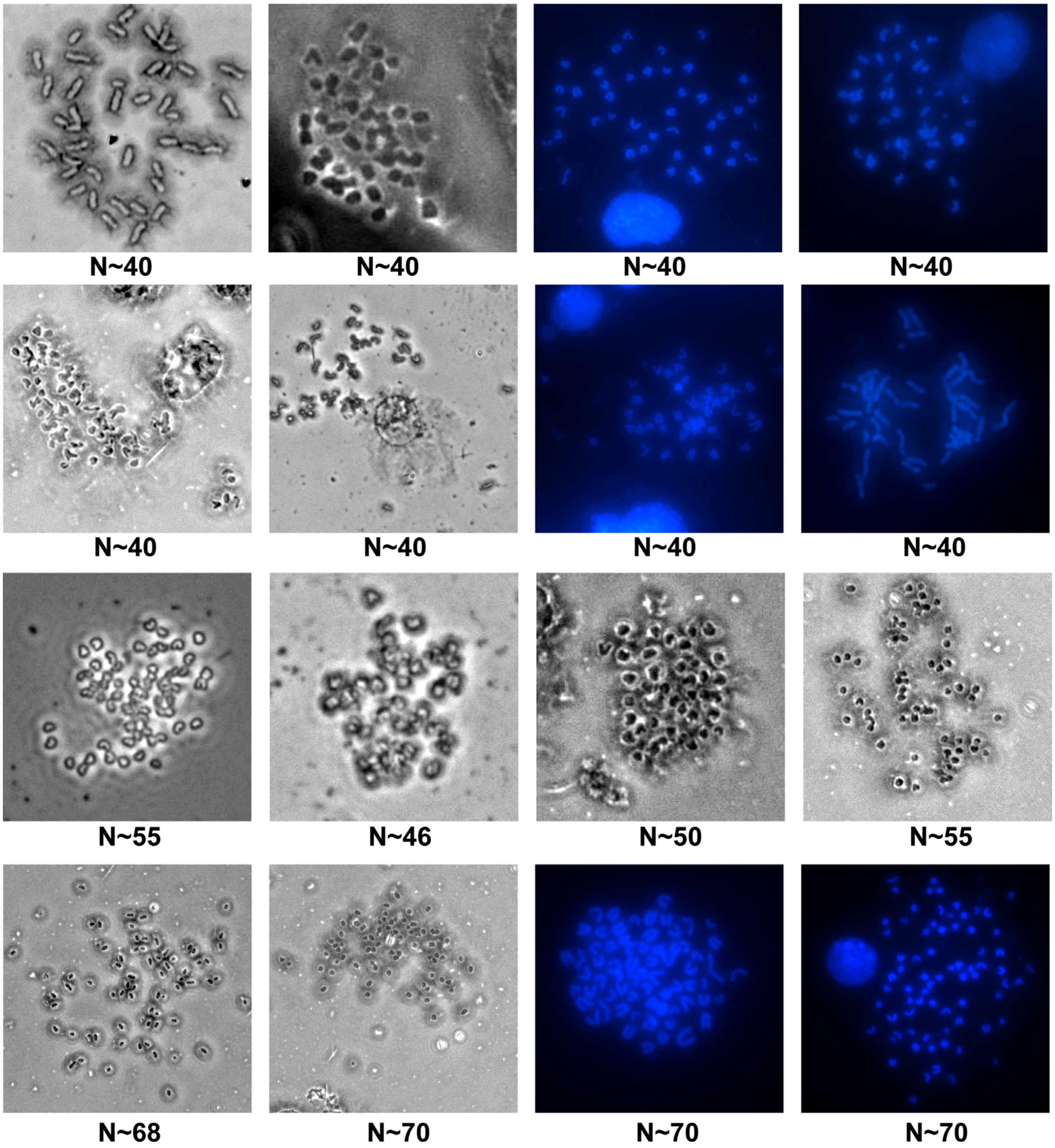

Supplementary Figure 5: Phase contrast images of metaphase nucleus spreads of tKM cells stained by Giemsa or DAPI. Numbers of chromosomes per metaphase nuclei indicated below images.

Supplementary Table 1: Cell and clone counts in reprogramming of various fibroblast lines by OKSM

Parent cell line	Day 0	Day 3		Day 14		
	Cell count per plate, $\times 10^{-3}$	Cell count per plate, $\times 10^{-3}$	Cell count per plate at transfer, $\times 10^{-3}$	Average numbers of AP positive clones		Average numbers of Nanog positive clones
				Compact clones per plate	Diffuse clones per plate	
MEF	50	168	56	110	10	181
			56	80	27	145
			56	105	10	125
NIH/3T3	50	375	125	61	50	0
			125	55	61	0
			125	20	70	0
STO	50	420	140	10	52	0
			140	5	20	0
			140	5	37	0
tKM	50	435	145	5	13	0
			145	0	10	0
			145	3	10	0

Supplementary Table 2: Chromosome counts in tKM cells

Chromosome numbers in tKM cells	Nuclei counts
N~40	23
N~60–75	11
N~30–36	3
N~46–55	4
